# Perceptions of Female Abdominal Muscle Definition: A Crowdsourced Analysis of Contour Preferences

**DOI:** 10.1093/asjof/ojag083

**Published:** 2026-05-13

**Authors:** Sahejbir S Bhatia, Cyrus Steppe, Amor Niksic, Y Edward Wen, William O W Layt, Al Aly

## Abstract

**Background:**

An athletic depiction of the female physique has risen in popularity recently, mirrored by a surge in techniques such as high-definition liposuction to achieve increased abdominal definition. Nonetheless, the perceived ideal degree of female abdominal etching remains unreported.

**Objectives:**

The objective of this study was to discern a diverse population's preferences for varying degrees of female abdominal etching and to understand demographic trends.

**Methods:**

A photo-realistic rendering of a female trunk displayed 3 levels of abdominal etching: basic, moderate, and extreme contour. Participants rated each image individually on a 7-point Likert scale and ranked the 3 images side-by-side from least to most attractive on Amazon Mechanical Turk, a crowdsourcing platform.

**Results:**

Nine hundred seventy-six responses were analyzed. Basic contour received the highest attractiveness median (interquartile range) score of 6.0 (2.0), moderate contour at 5 (2.0), and extreme contour at 4 (4.0) (*P* < .001). Compared with extreme contour, respondents had 4.33 times the odds of rating basic contour a 7.0 (*P* < .001). Compared with extreme contour, respondents had 14.6 times the odds of ranking basic contour as the most attractive (*P* < .001). Compared with moderate contour, respondents had 39.2 times the odds of ranking extreme contour least attractive (*P* < .001). Overall, 73% found basic contour most attractive, 11% found moderate contour most attractive, and 16% found extreme contour most attractive (*P* < .001).

**Conclusions:**

Respondents across demographics viewed less contoured female abdomens as more attractive than highly contoured abdomens.

**Level of Evidence: 5 (Therapeutic):**

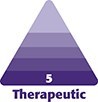

Artistic depictions over time have depicted the male body as sculpted and toned with noticeable muscle definition, but there has been a near complete absence of artistic portrayals presenting the female body in a similar manner. A notable exception is Michaelangelo's depiction of the female form on the ceiling of the Sistine Chapel, where females are essentially depicted as male bodies with breasts. This choice is likely due to the limited availability of female models in the 16th century rather than a reflection of social preferences.^1-3^ More recently, a departure from these modern societal norms may be attributed to the increasing recognition of the female athletic physique as more appealing than the historical beauty ideals that may be gleaned from traditional artwork. Nonetheless, the social perception of the ideal female form is diverse, with factors such as age, sex, sexuality, and ethnicity theorized to affect abdominal shape and contour preferences.^4,5^

These cultural trends in ideal body contour may be reflected in the popularity of liposuction to achieve a well-defined abdominal aesthetic. Abdominal sculpting goals are often subjective and dependent on the taste of the surgeon and the patient. A localized, superficial approach to liposuction dates to the early 1980s, with significant progression of liposuction technique occurring since then.^6,7^ The advent high-definition (HD) liposuction instigated a paradigm shift in the world of body contouring, where the methodology and technical implementation of artistic principles cater to each patient's body style and goals while systematically determining the appropriate degree of sculpting.^8^ Numerous surgeons have advanced HD liposuction to its current state of practice, with Hoyos being prominent in the field. He formulated the “BMX” protocol, which defines the body types he recommends pursuing, including basic (B), moderate (M), or extreme (X) etching to produce a desired level of muscle definition.^8^ Notably, others have contributed to this space with standardized guides for liposuction in large patient cohorts or have demonstrated reproducible results with power-assisted liposuction.^9,10^

With respect to the specific levels of abdominal etching, basic-definition liposuction involves the definition of large muscles and the reduction of adipose tissue in the abdomen. In Dr. Hoyos's practice, female patients commonly request this type of definition.^11^ Moderate-definition liposuction, the most requested level by men, enhances muscles that are often difficult to define through exercise alone, such as the obliques and transverse insertions of the rectus abdominis.^11^ Extreme-definition liposuction focuses on smaller muscle groups not mentioned previously, such as the serratus anterior. The goal of this liposuction treatment is to expose lean muscle mass and to sharpen muscle edges.^11^ Consequently, patients presenting for this procedure are typically leaner and fitter, as high-definition liposuction is not the ideal initial treatment choice for individuals with significant adiposity or large body habitus. Accordingly, the population undergoing this procedure represents a relatively selective subset of patients seeking body contouring.

The aim of this study is to explore and quantify the public's affinity for different degrees of female abdominal definition. Given the subjectivity of perceived attractiveness, crowdsourcing was used to obtain opinions of a large and diverse sample of respondents to identify trends in preferences for female abdominal definition.

## METHODS

**Figure 1.**
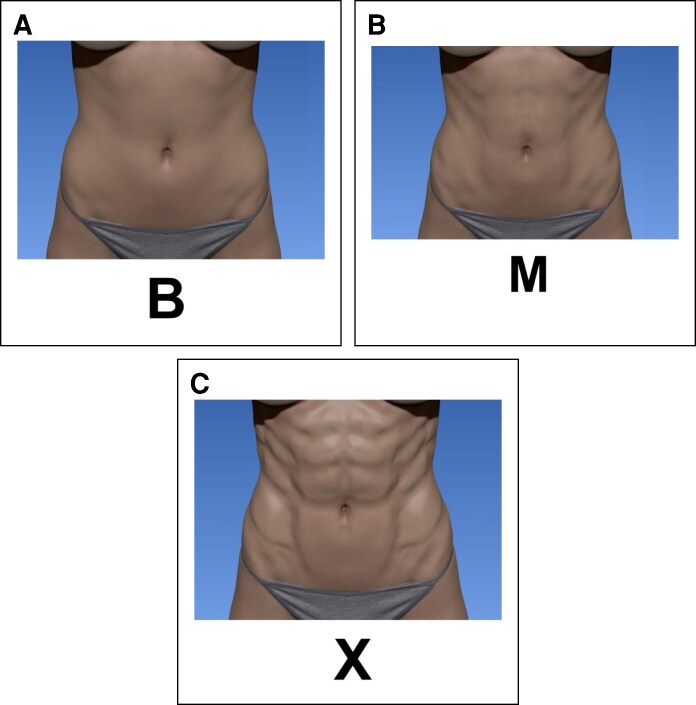
(A) One of the 3 digitally altered 3D renderings respondents were presented simultaneously to comparatively rank or individually in a random order to rate on a scale of 1 to 7. B represents basic contour. (B) One of the 3 digitally altered 3D renderings respondents were presented simultaneously to comparatively rank or individually in a random order to rate on a scale of 1 to 7. M represents moderate contour. (C) One of the 3 digitally altered 3D renderings respondents were presented simultaneously to comparatively rank or individually in a random order to rate on a scale of 1-7. X represents extreme contour.

A photo-realistic 3-dimensional full-body female scan was purchased from 3dscanstore.com. The scan was middle-aged, Caucasian, and between 20 and 23 BMI. The scan was then converted to a modifiable mesh, a 3D canvas that an artist can modify, and imported into the 3D rendering program Zbrush, a commonly used program for cinematic and video game character design (Maxon, Bad Homburg, Germany). The female mesh was then sculpted to emulate the 3 degrees of etching explained by Hoyos based on fitness and bodybuilding models (Figure 1A-C). Computer models were used to standardize all possible variables so that only the degree of etching was different between the images, which could not be achieved using actual postoperative patient photos. Etching attention was focused on the rectus abdominis, obliques, and serratus anterior. Each sculpt was then rendered on a blue background with identical lighting and cropped at the level of the mons pubis and below the nipple-areolar complex to insinuate a female form. In total, 3 abdomen types were created, ordered from least to most etching: basic, moderate, and extreme contour. The images were reviewed by a plastic surgeon before being used in the crowdsourcing survey.

A survey displaying the 3 images was created to collect crowdsourced preferences toward the degree of etching. Survey respondents viewed the images in a random order and were asked to rate the aesthetic appeal of the 3 images on a Likert scale ranging from 1 to 7, with 7 being the most attractive. Respondents also ranked the 3 images side by side from most to least attractive. The survey collected respondent demographics, including age, sex, sexuality, and ethnicity. This questionnaire was then uploaded to Amazon Mechanical Turk (MTurk), a crowdsourcing survey platform (Amazon, Seattle, WA). MTurk has been employed in numerous studies pertaining to aesthetics because it gathers data from large and diverse sample pools. Once a participant chooses to initiate the survey, they are directed to our survey platform to complete the questionnaire. MTurk employs quality control measures to reduce automated responses on the platform itself. Participant responses were used in the analysis if submission codes were accurately entered following completion of the survey. Submission codes are generated if the participant entered all demographic information, rankings, and ratings of the images. Four participants were excluded due to inaccurate input of submission codes. The survey was active from March 6, 2023 to March 10, 2023.

### Statistical Analysis

Continuous variables were reported as means with standard deviations or median [interquartile range (IQR)] as appropriate. For comparisons between numerical attractiveness ratings, Kruskal–Wallis or ANOVA tests were utilized as appropriate. For comparisons between categorical attractiveness rankings, chi-square or Fisher's exact tests were conducted as appropriate, with exact methods being used when expected cell counts were less than 5 for small subgroups. Shapiro–Wilk statistics were performed for normality checking. Post hoc pairwise comparisons were adjusted using the Bonferroni correction for multiple comparisons. For numerical attractiveness ratings and categorical attractiveness rankings, logistic regressions were used to calculate odds ratios (ORs) with 95% CIs. All data were analyzed at a significance level of 0.05 using Statistical Product and Service Solutions (SPSS) Statistics 25 (Armonk, New York).

## RESULTS

A total of 976 respondents were included in statistical analysis with 75% of respondents being from the USA. Age groups included 18 to 29 (238 respondents, 24%), 30 to 39 (398 respondents, 41%), 40 to 49 (191 respondents, 20%), 50 to 59 (91 respondents, 9%), 60 to 69 (44 respondents, 5%), and 70+ (14 respondents, 1%). Five hundred eighty-one respondents were female (60%), 376 were male (39%), and 19 were neither (2%). Sexual orientation groups included bisexual (181 respondents, 19%), heterosexual (691 respondents, 71%), homosexual (68 respondents, 7%), and other (36 respondents, 4%). Ethnic groups included Black (56 respondents, 6%), Caucasian (627 respondents, 64%), East Asian and Pacific Islander (40 respondents, 4%), Hispanic (77 respondents, 8%), Middle Eastern (9 respondents, 1%), Native American (63 respondents, 6%), and South Asian (104 respondents, 11%) (Table 1).

**Table 1.** Patient Demographics and Numerical Attractiveness Ratings

Respondent demographics	Respondents (*n*)	Rating of basic contour (median [IQR])	Rating of moderate contour (median [IQR])	Rating of extreme contour (median [IQR])	*P*-value
All	976	6.0 [2.0]	5.0 [2.0]	4.0 [4.0]	<.001*
Age (years)					
18-29	238	6.0 [2.0]	5.0 [2.0]	5.0 [3.0]	<.001*
30-39	398	6.0 [2.0]	5.0 [2.0]	4.0 [2.0]	<.001*
40-49	191	6.0 [2.0]	5.0 [2.0]	3.0 [3.0]	<.001*
50-59	91	6.0 [2.0]	5.0 [2.0]	3.0 [4.0]	<.001*
60-69	44	6.0 [2.0]	5.0 [2.0]	4.0 [2.0]	<.001*
70+	14	7.0 [2.0]	4.5 [1.0]	2.0 [3.0]	<.001*
Sex					
Female	581	6.0 [2.0]	5.0 [2.0]	4.0 [4.0]	<.001*
Male	376	6.0 [2.0]	5.0 [2.0]	4.0 [2.0]	<.001*
Other	19	6.0 [3.0]	5.0 [3.0]	4.0 [3.0]	.124
Sexuality					
Bisexual	181	6.0 [2.0]	5.0 [2.0]	5.0 [2.0]	<.001*
Heterosexual	691	6.0 [2.0]	5.0 [2.0]	4.0 [3.0]	<.001*
Homosexual	68	6.0 [2.0]	5.0 [2.0]	5.0 [3.0]	<.001*
Other	36	6.0 [2.0]	5.0 [2.0]	4.0 [4.0]	.051
Ethnicity					
Black	56	6.0 [2.0]	5.0 [2.0]	5.0 [3.0]	<.001*
Caucasian	627	6.0 [2.0]	5.0 [2.0]	4.0 [3.0]	<.001*
East Asian and Pacific Islander	40	6.0 [3.0]	5.0 [2.0]	4.0 [2.0]	.002*
Hispanic	77	6.0 [1.0]	5.0 [3.0]	5.0 [3.0]	<.001*
Middle Eastern	9	6.0 [1.0]	5.0 [2.0]	3.0 [4.0]	.019*
Native American	63	5.0 [1.0]	5.0 [2.0]	6.0 [2.0]	.473
South Asian	104	5.0 [2.0]	5.0 [2.0]	4.0 [3.0]	<.001*

^*^Statistically significant.

### Numerical Rating

#### Overall Analysis

Across all demographics, respondents strongly favored basic contour more than moderate contour which was favored more than extreme contour. There was a significant difference in numerical attractiveness rating between the abdomens with a median [IQR] rating of 6.0 [2.0] for basic contour, 5.0 [2.0] for moderate contour, and 4.0 [4.0] for extreme contour (*P* < .001) (Table 1).

#### Age Group

Attractiveness ratings for the degrees of etching were significantly different across age groups, with basic contour rated the highest, followed by moderate contour, and extreme contour rated the lowest (*P* < .001). For all ages under 70, the rating for basic contour was 6.0 [2.0] and moderate contour was 5.0 [2.0] (*P* < .001). For respondents over 70, the rating for the basic contour was 7.0 [2.0] and moderate contour was 4.5 [1.0] (*P* < .001). The rating for extreme contour for each age group was 18 to 29 (5.0 [3.0]), 30 to 39 (4.0 [2.0]), 40 to 49 (3.0 [3.0]), 50 to 59 (3.0 [4.0]), 60 to 69 (4.0 [2.0]), and over 70 (2.0 [3.0]) (*P* < .001) (Table 1).

#### Sex Analysis

The ratings between each of the 3 levels of etching were significantly different for male respondents and for female respondents, with basic contour rated the highest at 6.0 [2.0] by both groups, followed by moderate contour rated 5.0 [2.0] by both groups, and extreme contour rated the lowest at 4.0 [4.0] for females and 4.0 [2.0] for males (both *P* < .001). Patients who reported being neither male nor female rated basic contour 6.0 [3.0], moderate contour 5.0 [3.0], and extreme contour 4.0 [3.0] (*P* = .124) (Table 1).

#### Sexuality

Bisexual, heterosexual, homosexual, and other respondents all ranked basic contour 6.0 [2.0] and moderate contour at 5.0 [2.0]. The ratings for extreme contour were 5.0 [2.0] (bisexual), 4.0 [3.0] (heterosexual), 5.0 [3.0] (homosexual) (all *P* < .001), and 4.0 [3.0] (other) (*P* = .051) (Table 1).

#### Ethnicity

Black respondents rated basic, moderate, and extreme abdomen contours 6.0 [2.0], 5.0 [2.0], and 5.0 [3.0], respectively (*P* < .001). Caucasian respondents rated basic, moderate, and extreme abdomen contours at 6.0 [2.0], 5.0 [2.0], and 4.0 [3.0], respectively (*P* < .001). East Asian and Pacific Islander respondents rated basic, moderate, and extreme abdomen contours at 6.0 [3.0], 5.0 [2.0], and 4.0 [2.0], respectively (*P* = .002). Hispanic respondents rated basic, moderate, and extreme abdomen contours at 6.0 [1.0], 5.0 [3.0], and 5.0 [3.0], respectively (*P* < .001). Middle Eastern respondents rated basic, moderate, and extreme abdomen contours at 6.0 [1.0], 5.0 [2.0], and 3.0 [4.0], respectively (*P* = .019). Native American respondents rated basic, moderate, and extreme abdomen contours at 5.0 [1.0], 5.0 [2.0], and 6.0 [2.0], respectively (*P* = .473). South Asian respondents rated basic, moderate, and extreme abdomen contours at 5.0 [2.0], 5.0 [2.0], and 4.0 [3.0], respectively (*P* < .001) (Table 1).

### Attractiveness Ranking

#### Overall Analysis

Categorical attractiveness ranking between the abdomens differed significantly with 73% finding basic contour most attractive, 11% finding moderate contour most attractive, and 16% finding extreme contour the most attractive (all *P* < .001). Moreover, 16% found basic contour least attractive, 8% found moderate contour least attractive, and 77% found extreme contour least attractive (all *P* < .001). Across age, sex, sexuality, and ethnicity, respondents collectively ranked basic contour as the most attractive, moderate contour as second most attractive, and extreme contour as least attractive (all *P* < .001) (Supplemental Table 1).

#### Odds Ratios

Attractiveness numerical rating: compared with extreme contour, respondents had 4.3 times the odds of rating basic contour 7.0 (*P* < .001) and 1.2 times the odds of rating moderate contour a 7.0 (*P* = .193). Compared to moderate contour, respondents had 3.6 times the odds of rating basic contour 7.0 (*P* < .001) and 0.84 times the odds of rating extreme contour 7.0 (*P* = .193) (Tables 2, 3).

**Table 2.** Odds Ratios for Attractiveness Ratings by Abdomen Contour Type Compared With Extreme Contour

Abdomen type	Rating	Odds ratio^a^	95% CI	*P*-value
Basic contour				
	1	0.03	[0.01, 0.09]	<.001*
	2	0.06	[0.03, 0.12]	<.001*
	3	0.17	[0.12, 0.25]	<.001*
	4	0.47	[0.35, 0.63]	<.001*
	5	1.27	[1.01, 1.6]	.048*
	6	2.89	[2.30, 3.62]	<.001*
	7	4.33	[3.42, 5.49]	<.001*
Moderate contour				
	1	0.07	[0.03, 0.14]	<.001*
	2	0.18	[0.12, 0.27]	<.001*
	3	0.58	[0.44, 0.75]	<.001*
	4	1.16	[0.91, 1.47]	.226
	5	2.01	[1.61, 2.51]	<.001*
	6	2.28	[1.81, 2.87]	<.001*
	7	1.2	[0.91, 1.57]	.193

^a^Compared with extreme contour.

^*^Statistically significant.

**Table 3.** Odds Ratios for Attractiveness Ratings by Abdomen Contour Type Compared With Moderate Contour

Abdomen type	Rating	Odds ratio^a^	95% CI	*P*-value
Basic contour				
	1	0.50	[0.15, 1.66]	.256
	2	0.35	[0.17, 0.70]	<.001*
	3	0.30	[0.21, 0.45]	<.001*
	4	0.41	[0.31, 0.54]	<.001*
	5	0.63	[0.51, 0.78]	<.001*
	6	1.27	[1.04, 1.54]	<.001*
	7	3.62	[2.89, 4.54]	<.001*
Extreme contour				
	1	14.59	[7.07, 30.10]	<.001*
	2	5.58	[3.75, 8.34]	<.001*
	3	1.73	[1.33, 2.26]	<.001*
	4	0.86	[0.68, 1.19]	.226
	5	0.50	[0.40, 0.62]	<.001*
	6	0.44	[0.35, 0.55]	<.001*
	7	0.84	[0.64, 1.09]	.193

^a^Compared with moderate contour.

^*^Statistically significant.

Attractiveness comparative ranking: compared with extreme contour, respondents had 14.6 times the odds of ranking basic contour as the most attractive (*P* < .001) and 0.66 times the odds of ranking moderate contour as the most attractive (*P* = .002). Compared to moderate contour, respondents had 22.3 times the odds of ranking basic contour the most attractive and 1.52 times the odds of ranking extreme contour the most attractive (both *P* < .001). Compared to moderate contour, respondents had 39.2 times the odds of ranking extreme contour the least attractive (Tables 4, 5).

**Table 4.** Odds Ratios for Attractiveness Ranking by Abdomen Contour Type Compared With Extreme Contour

Abdomen type	Attractiveness ranking	Odds ratio^a^	95% CI	*P*-value
Basic contour				
	Most attractive	14.62	[11.70, 18.27]	<.001*
	Second most attractive	1.48	[1.09, 2.02]	<.001*
	Least attractive	0.06	[0.05, 0.07]	<.001*
Moderate contour				
	Most attractive	0.66	[0.50, 0.86]	.002*
	Second most attractive	52.41	[39.4, 69.72]	<.001*
	Least attractive	0.03	[0.02, 0.04]	<.001*

^a^Compared with extreme contour.

^*^Statistically significant.

**Table 5.** Odds Ratios for Attractiveness Ranking by Abdomen Contour Type Compared With Moderate Contour

Abdomen type	Attractiveness ranking	Odds ratio^a^	95% CI	*P*-value
Basic contour				
	Most attractive	22.25	[17.4, 28.45]	<.001*
	Second most attractive	0.03	[0.02, 0.04]	<.001*
	Least attractive	2.25	[1.68, 3.01]	<.001*
Extreme contour				
	Most attractive	1.52	[1.17, 1.98]	.002*
	Second most attractive	0.02	[0.01, 0.03]	<.001*
	Least attractive	39.19	[29.67, 51.76]	<.001*

^a^Compared with moderate contour.

^*^Statistically significant.

## DISCUSSION

The field of high-definition liposuction has evolved over several decades through the work of many groups. Currently, a prominent figure in the field is Alfredo Hoyos, who introduced the Basic/Moderate/Xtreme (BMX) protocol, providing a structured approach to attaining distinct levels of etching for certain levels of muscle definition.^8^ Others have developed stepwise guidelines for achieving reproducible results and minimizing complications based on experience from high-definition liposuction in large patient cohorts.^9,10^ In an evolving social landscape where societal preferences may be shifting toward an athletic female physique,^12^ high-definition liposuction is emerging as an increasingly popular avenue for achieving this coveted “ideal” abdominal aesthetic. While the BMX protocol has directed surgeons in tailoring treatments for different body conditions, there is a lack of reporting on the perception of various degrees of abdominal etching by the larger population.

The present study examined the perceived attractiveness of the female abdomen displayed in 3 degrees of definition of female abdominal contour. By utilizing a photo-realistic 3D model and employing crowdsourcing methods to maintain a diverse sample population, the study evaluated preferences for abdominal definition across different age groups, sexes, sexualities, and ethnicities. Understanding societal preferences for abdominal aesthetics may be useful in supplementing aesthetic and body contouring surgeons with objective information when discussing subjective patient goals and setting expectations.

For attractiveness rankings, participants across demographics consistently rated the female abdomen with basic contour as the most attractive, followed by the abdomen with moderate contour, and finally the abdomen with extreme contour. These findings suggest a strong preference for less defined abdomens, despite the cultural shift toward athletic physiques.^13,14^

Further evaluating the impact of age, the basic contour consistently received the highest numerical ratings, indicating a cross-generational affinity for minimally contoured abdomens. Conversely, it is evident that each generation may still have differing attitudes toward abdominal contours and associated desirability. Older participants (60+) demonstrated a stronger preference for basic contour abdomens than younger age groups did. This preference may be attributed to a conditioned preference for a less muscular female habitus in congruence with historical trends surrounding female body types.^15^ In contrast, participants aged 18 to 29 more frequently rated the extremely contoured abdomen as being more attractive than the other age groups did. Younger age groups may have a greater desire to achieve exceptional and attention-demanding results in various aspects of life, including physical appearance.^16^ Extreme body contour represents an intensified level of definition, which may be viewed as a desirable achievement for those seeking a salient presence. Existing literature suggests that younger generations are more inclined toward conforming to contemporary trends, including those related to physical appearance.^17^ In recent years, this trend has promoted highly defined and athletic bodies as the feminine beauty standard,^14^ thus creating high exposure to such body types.

The prevalence of more athletic female figures in sports and entertainment may also contribute to this phenomenon. High societal exposure affecting perceived attractiveness may be explained by the principle of cognitive averaging, which posits that repeated mainstream exposure to initially unusual or atypical concepts may eventually normalize that concept, making it no longer unusual.^18-21^ Cognitive averaging may be a key pillar in discerning the preferences between older and younger raters, who likely have drastically differing levels of popular culture exposure to highly defined female abdomens.^18-21^

Sexuality and ethnicity also exhibited distinct patterns. For instance, bisexual participants demonstrated no consistent preference between images compared to other sexual orientations. In terms of ethnicity, there were differences in attractiveness ratings among groups, which may evidence the diverse cultural elements influencing perceptions of beauty.^22^ Such cultural elements include features like body proportions, dimensions, and contours.^22-24^ Moreover, the ongoing process of globalization and cultural exchange fosters the blending and redefinition of beauty norms. This process is likely nonuniform across all ethnic groups, which leads to variations in attractiveness standards based on the degree of exposure to global or regional beauty trends. In this cohort, 75% of respondents were from the United States, and while there is a diversity of cultural norms within the country, the findings in this study largely represent a Western perspective on abdominal contour preferences.

Side-by-side ranking of abdominal contours furthered the notion that basic contour is perceived as the most attractive, followed by moderate contour then extreme contour. These data suggest that the preference is not only absolute but also comparative. Both cultural influences and psychological elements are implicated in these findings. Historically, many cultures have associated softer and less defined body contours with femininity and beauty.^2^ This prevailing view has been cultivated by the interplay of art, media, and societal norms.^2^ The observed widespread preference for the female body with basic abdominal etching appears to align with these cultural ideas of beauty preserving a more natural contour. The body with moderate abdominal etching follows this trend, offering some definition while still maintaining some sense of approachability and comfort.

The consistently lower attractiveness rating for the abdomen with extreme etching may be multifactorial. First, it is possible that the extreme level of definition deviates from the traditional softness associated with feminine beauty.^23^ Second, extreme etching has been shown to evoke associations with intense fitness regimes or potential body image concerns, affecting the perceived attractiveness negatively. For example, exposure to images of fit and thin models is associated with greater physique anxiety, decreased body satisfaction, negative mood, and lowered self-esteem.^25-27^ There is also growing awareness and backlash against unrealistic beauty standards perpetuated by media and advertising.^28,29^ Moreover, a shifting societal context advocating inclusivity and body positivity emphasizes accommodating variations in body shapes rather than conforming to any particular ideal.^30^ Nonetheless, the cultural shift toward athletic physiques has steered plastic surgeons toward emphasizing muscularity and definition in cosmetic liposuction procedures. The findings indicate a persistent preference for a less-defined abdominal appearance. Notably, while extreme contour was overall less favored and most often ranked as the least attractive, it was still chosen as most attractive slightly more frequently than moderate contour was (16% for extreme contour and 11% for moderate contour). This deviation from the overall trend suggests a degree of polarization in perceptions of extreme contour rather than a consistent negative perception.

In extension, the strong cross-demographic preference for the least defined female abdomen is possibly a manifestation of more deep-seated evolutionary biological principles based on the work of Charles Darwin, Gregor Mendel, and Richard Dawkins, which suggest that the genetic implications of particular traits fundamentally drive the success of those traits over time.^4,31-33^ These principles may serve as a different perspective by which human behavior and preferences can be interpreted. For traits such as limited abdominal definition to persist through time in human populations, the trait likely must have provided a survival or reproductive advantage. Specifically, in prehistoric societies when humans were nomadic and relied upon hunting and gathering, a highly lean appearing female habitus would probabilistically occur in the context of a limited caloric availability caused by circumstances such as a metabolically expensive disease burden or limited nutritional access. In this study, the extremely etched contour displayed a level of definition that would be expected in a female with 12% to 15% body fat,^32,34,35^ and it has been demonstrated that body fat percentages less than 18% are significantly associated with a greater risk of anovulation, menstrual irregularities, subfertility, and hypothalamic–pituitary–ovarian axis dysfunction.^32,33,36,37^ Consequently, female sexual partners with such a body habitus in these early societies would be less likely to successfully transmit their genes to future generations. Conversely, females with signs of fertility and caloric availability, which is better represented by the basic contour, would not necessarily be faced with similar challenges, thereby increasing the chance that their genetics would prevail in the population. Importantly, it should be noted that such explanations are theoretical and are based on evolutionary theory rather than being a definitive causal explanation of the findings. The findings in this study are not applicable to assessing the genetic or fertility implications of any abdomen.

From another perspective, it is important to recognize that the trend of offering more definition through liposuction may also relate to the marketability of change itself, irrespective of the actual nature of the change. Specifically, if the opportunity for a visible change in appearance can be marketed as desirable to patients, a certain proportion of patients may pursue the surgeon's suggestion without a true underlying desire for that particular change. In fact, it has been shown that cosmetic procedures are often pursued for reasons other than an underlying desire for a specified physical change.^38-40^ For example, for decades of the senior author's career, aggressively elevating the brows was presented to patients as attractive.^41-43^ Eventually, based on observation and systematic investigation of ideal brow position in the literature coupled with patient dissatisfaction, it became clear that not all brow elevations are good or ideal despite their popularity at the time. That is to say, just because something can be pursued does not mean it should be.

This study contained limitations. The use of crowdsourcing, while providing a large and diverse sample, may lack the nuance of face-to-face evaluations. With any surveying study, there are challenges in ensuring the quality of rater responses. We endeavored to collect as many high-quality responses as possible through the implementation of response filters, but thoughtful responses truly reflecting rater sentiment are not guaranteed. Additionally, this study primarily focused on a single 3D model based on a middle-aged Caucasian woman, which does not capture the full spectrum of body types and shapes. It is likely that manipulating other attributes of the model including skin color, laxity, congenital markings, stretch marks, areas of pigmentation, scars, among other skin or shape characteristics could influence the results. Conversely, it is important to note that increasing the representativeness of the abdomens increases the impact of confounding variables on the findings. Controlling all possible variables except for degree of etching as was done in the current study allows for maximizing internal validity of the findings such that the differences in perceived attractiveness are due to the degree of etching alone, though this reduces the external validity of the study. Moreover, it was not the intent of the authors to generalize these findings to the general population because high-definition liposuction is not the optimal procedure for addressing abdominal definition in patients with excess adiposity or large body habitus. Additionally, small sizes in some demographic subgroups may have limited power for subgroup comparisons and should be interpreted cautiously. In the future, our group is considering using different races and body types to determine the generalizability of our findings. The study did not explore the potential impact of other factors such as degree of cultural media exposure and personal experiences on participant preferences, which is challenging to devise and operationalize in a cross-cultural manner.

## CONCLUSIONS

Less contoured female abdomens had a greater appeal among respondents with the basic contour being rated more favorably than the moderate contour, which was rated more favorably than the extreme contour. There were several notable variations within this pattern among the youngest and oldest cohorts and among other demographic groups.

## Supplementary Material

ojag083_Supplementary_Data

## References

[1] Nochlin L . Why have there been No great women artists? In: Harrison C, Wood P, eds. Art in Theory 1900-2000: An Anthology of Changing Ideas. Blackwell; 1992:520–534.

[2] Parker R . The female body and body image: a historical perspective. In: Parker R, ed. Women, Doctors and Cosmetic Surgery: Negotiating the ‘Normal’ Body. Palgrave Macmillan UK; 2009:25–37.

[3] Michel E, Schröder KA, eds. Michelangelo and Beyond. Exhibition catalogue. Albertina Museum. Prestel Verlag; 2023. ISBN 9783791391168.

[4] Hart RJ . Physiological aspects of female fertility: role of the environment, modern lifestyle, and genetics. Physiol Rev. 2016;96:873–909. doi: 10.1152/physrev.00023.2015 27252278

[5] Kronenfeld LW, Reba-Harrelson L, Von Holle A, Reyes ML, Bulik CM. Ethnic and racial differences in body size perception and satisfaction. Body Image. 2010;7:131–136. doi: 10.1016/j.bodyim.2009.11.002 20096656 PMC3593344

[6] Sterodimas A, Boriani F, Magarakis E, Nicaretta B, Pereira LH, Illouz YG. Thirty four years of liposuction: past, present and future. Eur Rev Med Pharmacol Sci. 2012;16:393–406. PMID: 22530358. 22530358

[7] Willet JW, Alvaro AI, Ibrahim AK, Javed MU. A systematic review of efficacy and complications of high-definition liposuction. Plast Reconstr Surg. 2023;152:57–63. doi: 10.1097/PRS.0000000000010203 36728192

[8] Hoyos AE, Millard JA. VASER-assisted high-definition liposculpture. Aesthet Surg J. 2007;27:594–604. doi: 10.1016/j.asj.2007.08.007 19341688

[9] Saad AN, Pablo Arbelaez J, De Benito J. High definition liposculpture in male patients using reciprocating power-assisted liposuction technology: techniques and results in a prospective study. Aesthet Surg J. 2020;40:299–307. doi: 10.1093/asj/sjz218 31361804

[10] Athanasiou A, Siozou M, Maltzaris N, Neamonitou F, Rempelos G. A 7-step guide to high-definition liposuction. Aesthetic Plast Surg. 2022;46:2863–2879. doi: 10.1007/s00266-022-02965-w 35729373

[11] Hoyos AE, Perez ME, Domínguez-Millán R. Variable sculpting in dynamic definition body contouring: procedure selection and management algorithm. Aesthet Surg J. 2020;41:318–332. doi: 10.1093/asj/sjaa133 32455430

[12] Bozsik F, Whisenhunt BL, Hudson DL, Bennett B, Lundgren JD. Thin is in? Think again: the rising importance of muscularity in the thin ideal female body. Sex Roles. 2018;79:609–615. doi: 10.1007/s11199-017-0886-0

[13] Homan K . Athletic-ideal and thin-ideal internalization as prospective predictors of body dissatisfaction, dieting, and compulsive exercise. Body Image. 2010;7:240–245. doi: 10.1016/j.bodyim.2010.02.004 20226748

[14] Carrotte ER, Prichard I, Lim MS. Fitspiration” on social media: a content analysis of gendered images. J Med Internet Res. 2017;19:e95. doi: 10.2196/jmir.6368 28356239 PMC5390113

[15] Han C, Lei X, Yan P, Li X, Morrison ER. Age differences in preferences for body physique. Pers Individ Dif. 2021;181:111033. doi: 10.1016/j.paid.2021.111033

[16] McElhaney KB, Antonishak J, Allen JP. They like me, they like me not”: popularity and adolescents’ perceptions of acceptance predicting social functioning over time. Child Dev. 2008;79:720–731. doi: 10.1111/j.1467-8624.2008.01153.x 18489423 PMC3073367

[17] Chierchia G, Piera Pi-Sunyer B, Blakemore SJ. Prosocial influence and opportunistic conformity in adolescents and young adults. Psychol Sci. 2020;31:1585–1601. doi: 10.1177/0956797620957625 33226891 PMC7734552

[18] Baek J, Chong SC. Distributed attention model of perceptual averaging. Atten Percept Psychophys. 2020;82:63–79. doi: 10.3758/s13414-019-01827-z 31347018

[19] Bechler CJ, Levav J. Compatibility effects in the perception of dispersion. Cognition. 2022;225:105166. doi: 10.1016/j.cognition.2022.105166 35644092

[20] Deutsch R, Ebert J, Barth M, Roth J. Biased perception of distributions: anchoring, interpolation and smoothing as potential causes. Cognition. 2023;237:105448. doi: 10.1016/j.cognition.2023.105448 37229925

[21] Hadar B, Glickman M, Trope Y, Liberman N, Usher M. Abstract thinking facilitates aggregation of information. J Exp Psychol Gen. 2022;151:1733–1743. doi: 10.1037/xge0001126 34928684

[22] Voegeli R, Schoop R, Prestat-Marquis E, Rawlings AV, Shackelford TK, Fink B. Cross-cultural perception of female facial appearance: a multi-ethnic and multi-centre study. PLoS One. 2021;16:e0245998. doi: 10.1371/journal.pone.0245998 33481957 PMC7822532

[23] Dimitrov D, Kroumpouzos G. Beauty perception: a historical and contemporary review. Clin Dermatol. 2023;41:33–40. doi: 10.1016/j.clindermatol.2023.02.006 36878443

[24] Little AC, Jones BC, DeBruine LM. Facial attractiveness: evolutionary based research. Philos Trans R Soc Lond B Biol Sci. 2011;366:1638–1659. doi: 10.1098/rstb.2010.0404 21536551 PMC3130383

[25] Sabiston CM, Chandler K. Effects of fitness advertising on weight and body shape dissatisfaction, social physique anxiety, and exercise motives in a sample of healthy-weight females. J Appl Biobehav Res. 2009;14:165–180. doi: 10.1111/j.1751-9861.2010.00047.x

[26] Benton C, Karazsia BT. The effect of thin and muscular images on women's body satisfaction. Body Image. 2015;13:22–27. doi: 10.1016/j.bodyim.2014.11.001 25528369

[27] Tiggemann M, Zaccardo M. Exercise to be fit, not skinny”: the effect of fitspiration imagery on women's body image. Body Image. 2015;15:61–67. doi: 10.1016/j.bodyim.2015.06.003 26176993

[28] MacCallum F, Widdows H. Altered images: understanding the influence of unrealistic images and beauty aspirations. Health Care Anal. 2018;26:235–245. doi: 10.1007/s10728-016-0327-1 27432005 PMC6061013

[29] Duncan MC . The politics of women's body images and practices: Foucault, the panopticon, and shape magazine. J Sport Soc Issues. 1994;18:48–65. doi: 10.1177/019372394018001004

[30] Griffin M, Bailey KA, Lopez KJ. #BodyPositive? A critical exploration of the body positive movement within physical cultures taking an intersectionality approach. Front Sports Act Living. 2022;4:908580. doi: 10.3389/fspor.2022.908580 36299403 PMC9589104

[31] Jasienska G, Bribiescas RG, Furberg AS, Helle S, Núñez-de la Mora A. Human reproduction and health: an evolutionary perspective. Lancet. 2017;390:510–520. doi: 10.1016/S0140-6736(17)30573-1 28792413

[32] Hernaez A, Rogne T, Skåra KH, et al Body mass index and subfertility: multivariable regression and Mendelian randomization analyses in the Norwegian Mother, Father and Child Cohort Study. Hum Reprod. 2021;36:3141–3151. doi: 10.1093/humrep/deab224 34668019 PMC8600658

[33] Zhou J, Zhang Y, Teng Y, et al Association between preconception body mass index and fertility in adult female: a systematic review and meta-analysis. Obes Rev. 2024;25:e13804. doi: 10.1111/obr.13804 39054661

[34] Klungland Torstveit M, Sundgot-Borgen J. Are under- and overweight female elite athletes thin and fat? A controlled study. Med Sci Sports Exerc. 2012;44:949–957. doi: 10.1249/MSS.0b013e31823fe4ef 22089480

[35] van der Ploeg GE, Brooks AG, Withers RT, Dollman J, Leaney F, Chatterton BE. Body composition changes in female bodybuilders during preparation for competition. Eur J Clin Nutr. 2001;55:268–277. doi: 10.1038/sj.ejcn.1601154 11360131

[36] Lee IT, Barnhart KT, Hwang WT, et al Association between markers of female adiposity and live birth among patients undergoing fertility treatment or attempting unassisted conception. Hum Reprod. 2025;40:1671–1680. doi: 10.1093/humrep/deaf124 40605077 PMC12756995

[37] McKinnon CJ, Hatch EE, Rothman KJ, et al Body mass index, physical activity and fecundability in a North American preconception cohort study. Fertil Steril. 2016;106:451–459. doi: 10.1016/j.fertnstert.2016.04.011 27125230

[38] Javo IM, Sørlie T. Psychosocial characteristics of young Norwegian women interested in liposuction, breast augmentation, rhinoplasty, and abdominoplasty: a population-based study. Plast Reconstr Surg. 2010;125:1536–1543. doi: 10.1097/PRS.0b013e3181d5135a 20440172

[39] Maisel A, Waldman A, Furlan K, et al Self-reported patient motivations for seeking cosmetic procedures. JAMA Dermatol. 2018;154:1167–1174. doi: 10.1001/jamadermatol.2018.2357 30140900 PMC6233736

[40] Sarwer DB, Cash TF, Magee L, et al Female college students and cosmetic surgery: an investigation of experiences, attitudes, and body image. Plast Reconstr Surg. 2005;115:931–938. doi: 10.1097/01.prs.0000153204.37065.d3 15731697

[41] Coombs DM, Sinclair NR, Kochuba A, et al Practice patterns: an American Society of Plastic Surgeons (ASPS) member survey, 2000 and 2020-how much has brow lifting changed? Aesthet Surg J. 2023;44:1–8. doi: 10.1093/asj/sjad207 37409963

[42] Paul MD . The evolution of the brow lift in aesthetic plastic surgery. Plast Reconstr Surg. 2001;108:1409–1424. doi: 10.1097/00006534-200110000-00048 11604652

[43] Rohrich RJ, Cho MJ. Endoscopic temporal brow lift: surgical indications, technique, and 10-year outcome analysis. Plast Reconstr Surg. 2019;144:1305–1310. doi: 10.1097/PRS.0000000000006238 31764641

