# Supplementary material for: Perceptions of Female Abdominal Muscle Definition: A Crowdsourced Analysis of Contour Preferences
Source: Aesthet Surg J Open Forum. 2026 May 13;8:ojag083. doi: 10.1093/asjof/ojag083 (PMC13228994; doi:10.1093/asjof/ojag083)
Supplement: ojag083_Supplementary_Data [file ojag083_supplementary_data.docx]

**Supplemental Table 1. Respondent demographics and attractiveness ranking by abdomen contour**

| **Respondent Demographics** | **Respondents (n)** | **Ranking of Basic Contour** | | | **Ranking of Moderate Contour** | | | **Ranking of Extreme Contour** | | | **P-value** |
| --- | --- | --- | --- | --- | --- | --- | --- | --- | --- | --- | --- |
|  |  | **Most Attractive, n (%)** | **2nd Most Attractive, n (%)** | **Least Attractive, n (%)** | **Most Attractive, n (%)** | **2nd Most Attractive, n (%)** | **Least Attractive, n (%)** | **Most Attractive, n (%)** | **2nd Most Attractive, n (%)** | **Least Attractive, n (%)** |  |
| **All** | 976 | 715 (73.3%) | 107 (11.0%) | 154 (15.8%) | 107 (11.0%) | 794 (81.4%) | 75 (7.7%) | 154 (15.8%) | 75 (7.7%) | 747 (76.5%) | <0.001* |
| **Age (years)** |  |  |  |  |  |  |  |  |  |  |  |
| 18-29 | 238 | 144 (60.5%) | 32 (13.4%) | 62 (26.1%) | 29 (12.2%) | 178 (74.8%) | 31 (13.0%) | 65 (27.3%) | 28 (11.8%) | 145 (60.9%) | <0.001* |
| 30-39 | 398 | 293 (73.6%) | 50 (12.6%) | 55 (13.8%) | 56 (14.1%) | 310 (77.9%) | 32 (8.0%) | 59 (14.8%) | 28 (7.0%) | 311 (78.1%) | <0.001* |
| 40-49 | 191 | 157 (82.2%) | 11 (5.8%) | 23 (12.0%) | 16 (8.4%) | 170 (89.0%) | 5 (2.6%) | 18 (9.4%) | 10 (5.2%) | 163 (85.3%) | <0.001* |
| 50-59 | 91 | 74 (81.3%) | 8 (8.8%) | 9 (9.9%) | 10 (11.0%) | 77 (84.6%) | 4 (4.4%) | 7 (7.7%) | 6 (6.6%) | 78 (85.7%) | <0.001* |
| 60-69 | 44 | 33 (75.0%) | 6 (13.6%) | 5 (11.4%) | 6 (13.6%) | 35 (79.5%) | 3 (6.8%) | 5 (11.4%) | 3 (6.8%) | 36 (81.8%) | <0.001* |
| 70+ | 14 | 14 (100.0%) | 0 (0.0%) | 0 (0.0%) | 0 (0.0%) | 14 (100.0%) | 0 (0.0%) | 0 (0.0%) | 0 (0.0%) | 14 (100.0%) | <0.001* |
| **Sex** |  |  |  |  |  |  |  |  |  |  |  |
| Female | 581 | 414 (71.3%) | 69 (11.9%) | 98 (16.9%) | 70 (12.0%) | 466 (80.2%) | 45 (7.7%) | 97 (16.7%) | 46 (7.9%) | 438 (75.4%) | <0.001* |
| Male | 376 | 288 (76.6%) | 36 (9.6%) | 52 (13.8%) | 35 (9.3%) | 313 (83.2%) | 28 (7.4%) | 53 (14.1%) | 27 (7.2%) | 296 (78.7%) | <0.001* |
| Other | 19 | 13 (68.4%) | 2 (10.5%) | 4 (21.1%) | 2 (10.5%) | 15 (78.9%) | 2 (10.5%) | 4 (21.1%) | 2 (10.5%) | 13 (68.4%) | <0.001* |
| **Sexuality** |  |  |  |  |  |  |  |  |  |  |  |
| Bisexual | 181 | 109 (60.2%) | 19 (10.5%) | 53 (29.3%) | 18 (9.9%) | 146 (80.7%) | 17 (9.4%) | 54 (29.8%) | 16 (8.8%) | 111 (61.3%) | <0.001* |
| Heterosexual | 691 | 535 (77.4%) | 75 (10.9%) | 81 (11.7%) | 77 (11.1%) | 565 (81.8%) | 49 (7.1%) | 79 (11.4%) | 51 (7.4%) | 561 (81.2%) | <0.001* |
| Homosexual | 68 | 43 (63.2%) | 9 (13.2%) | 16 (23.5%) | 8 (11.8%) | 53 (77.9%) | 7 (10.3%) | 17 (25.0%) | 6 (8.8%) | 45 (66.2%) | 0.002* |
| Other | 36 | 28 (77.8%) | 4 (11.1%) | 4 (11.1%) | 4 (11.1%) | 30 (83.3%) | 2 (5.5%) | 4 (11.1%) | 2 (5.5%) | 30 (83.3%) | <0.001* |
| **Ethnicity** |  |  |  |  |  |  |  |  |  |  |  |
| Black | 56 | 42 (75.0%) | 8 (14.3%) | 6 (10.7%) | 9 (16.1%) | 41 (73.2%) | 6 (10.7%) | 5 (8.9%) | 7 (12.5%) | 44 (78.6%) | <0.001* |
| Caucasian | 627 | 483 (77.0%) | 62 (9.9%) | 82 (13.1%) | 63 (10.0%) | 524 (83.6%) | 40 (6.4%) | 81 (12.9%) | 41 (6.5%) | 505 (80.5%) | <0.001* |
| East Asian and Pacific Islander | 40 | 27 (67.5%) | 8 (20.0%) | 5 (12.5%) | 8 (20.0%) | 30 (75.0%) | 2 (5.0%) | 5 (12.5%) | 2 (5.0%) | 33 (82.5%) | <0.001* |
| Hispanic | 77 | 45 (58.4%) | 10 (13.0%) | 22 (28.6%) | 10 (13.0%) | 60 (77.9%) | 7 (9.1%) | 22 (28.6%) | 7 (9.1%) | 48 (62.3%) | <0.001* |
| Middle Eastern | 9 | 8 (88.9%) | 0 (0.0%) | 1 (11.1%) | 0 (0.0%) | 8 (88.9%) | 1 (11.1%) | 1 (11.1%) | 1 (11.1%) | 7 (77.8%) | <0.001* |
| Native American | 63 | 33 (52.4%) | 6 (9.5%) | 24 (38.1%) | 7 (11.1%) | 49 (77.8%) | 7 (11.1%) | 23 (36.5%) | 8 (12.7%) | 32 (50.8%) | <0.001* |
| South Asian | 104 | 77 (74.0%) | 13 (12.5%) | 14 (13.5%) | 10 (9.6%) | 82 (78.8%) | 12 (11.5%) | 17 (16.3%) | 9 (8.7%) | 78 (75.0%) | <0.001* |

*Statistically significant.
